# Microbially mediated carbon dioxide removal for sustainable mining

**DOI:** 10.1371/journal.pbio.3002026

**Published:** 2023-03-21

**Authors:** Jenine McCutcheon, Ian M. Power

**Affiliations:** 1 Department of Earth and Environmental Sciences, University of Waterloo, Waterloo, Canada; 2 Trent School of the Environment, Trent University, Peterborough, Canada; PLOS Biology, UNITED KINGDOM

## Abstract

Historically thought of as waste, mine tailings are an underutilized source of metals and carbon dioxide sink. This Perspective outlines how microbes can be used to help the mining industry transition to a more sustainable future.

## Teaser

The climate crisis and rising demand for critical minerals necessitate the development of novel carbon dioxide removal and ore processing technologies. Microbial processes can be harnessed to recover metals from and store carbon dioxide within mine tailings to transform the mining industry for a greener and more sustainable future.

The rise of vascular land plants (380–350 Ma) led to an immense transfer of carbon dioxide (CO_2_) from the atmosphere to the geosphere via photosynthesis and organic matter burial, leading to fossil fuel formation [1]. In a matter of centuries, humans have largely reversed this natural carbon storage through petroleum extraction and combustion, having emitted 2400 ± 240 Gt of CO_2_ to the atmosphere since 1850 [2]. Anthropogenic CO_2_ and other greenhouse gases (GHG) have already caused a 1.1°C increase above pre-industrial global surface temperatures [2]. The most recent Intergovernmental Panel on Climate Change (IPCC) report indicates that even if warming can be limited to 1.5°C, we will face multiple climate impacts in the near-term [2]. Therefore, we must urgently phase out fossil fuels and recapture the excess CO_2_ we have already emitted to the atmosphere. Commensurate to the climate crisis is the rising demand for critical minerals with the development of green energy and technologies, including solar panels, electric vehicles, and batteries. Consequently, society will demand greater mineral resources while expecting the mining sector to achieve carbon neutrality.

While the IPCC 2022 Mitigation of Climate Change report outlines several carbon dioxide removal (CDR) pathways [2], one option that warrants greater attention is the consumption of CO_2_ through enhanced weathering and carbonation of minerals at mine sites. CO_2_ reacts with major rock-forming minerals to release dissolved cations (Ca^2+^, Mg^2+^, K^+^) and inorganic carbon (HCO_3_^-^), which are eventually transported to the oceans, storing carbon as alkalinity and solid carbonate minerals (e.g., CaCO_3_) such as those in corals and limestone [3]. Weathering is the breakdown of rocks through chemical and physical processes and is an important regulator of atmospheric CO_2_. Silicate and carbonate rock weathering naturally consumes an estimated 1.2 to 3.0 Gt CO_2_/yr globally, a number that will increase in response to warmer temperatures and higher CO_2_ concentrations [4,5]. Microbes contribute to weathering by attaching to mineral surfaces, generating rock-dissolving acids through fermentation and respiration, releasing chelating compounds, and using minerals as starting materials for energetically favourable redox reactions. Different microbes target different minerals, and their growth depends on environmental and geochemical parameters such as temperature, salinity, oxygenation, pH, and water availability. Additionally, some microbes accelerate the formation of secondary minerals using the by-products of mineral weathering as reactants. Secondary minerals can include carbonate minerals (i.e., microbial mineral carbonation), the formation of which transfers CO_2_ from the atmosphere back to the geosphere for long-term storage [6].

Natural biogeochemical weathering processes are too slow to mitigate anthropogenic CO_2_ emissions on the necessary timescales. However, there is potential to accelerate CDR using fine-grained rock powders, such as mine tailings, which are mineral wastes generated from ore processing. A variety of tailing types, produced at a rate of approximately 13 Gt/yr globally (predominantly from Cu, Fe, and Au production), have been evaluated for their CDR potential, with alkaline tailings (approximately 420 Mt/yr) from diamond, nickel, cobalt, and other metal mines being best suited for enhanced weathering and mineral carbonation [6,7]. Weathering of tailing minerals consumes CO_2_ and can be accelerated using microbial processes that produce fluids for mineral carbonation reactions. Bioleaching is an established mining practice for increasing mineral dissolution rates and can be tuned for different ores, including those that generate alkaline tailings [8,9]. More ore-specific research is needed to improve microbial community selection, predictions of mineral weathering rates, and best practices for large-scale implementation. A benefit of accelerating mineral weathering in historic stockpiles is that tailings at many legacy facilities contain low concentrations of critical minerals, including nickel and cobalt, that were not previously worth extracting [10]. Bioleaching can recover these metals to help meet increasing global demands.

**Fig 1:**
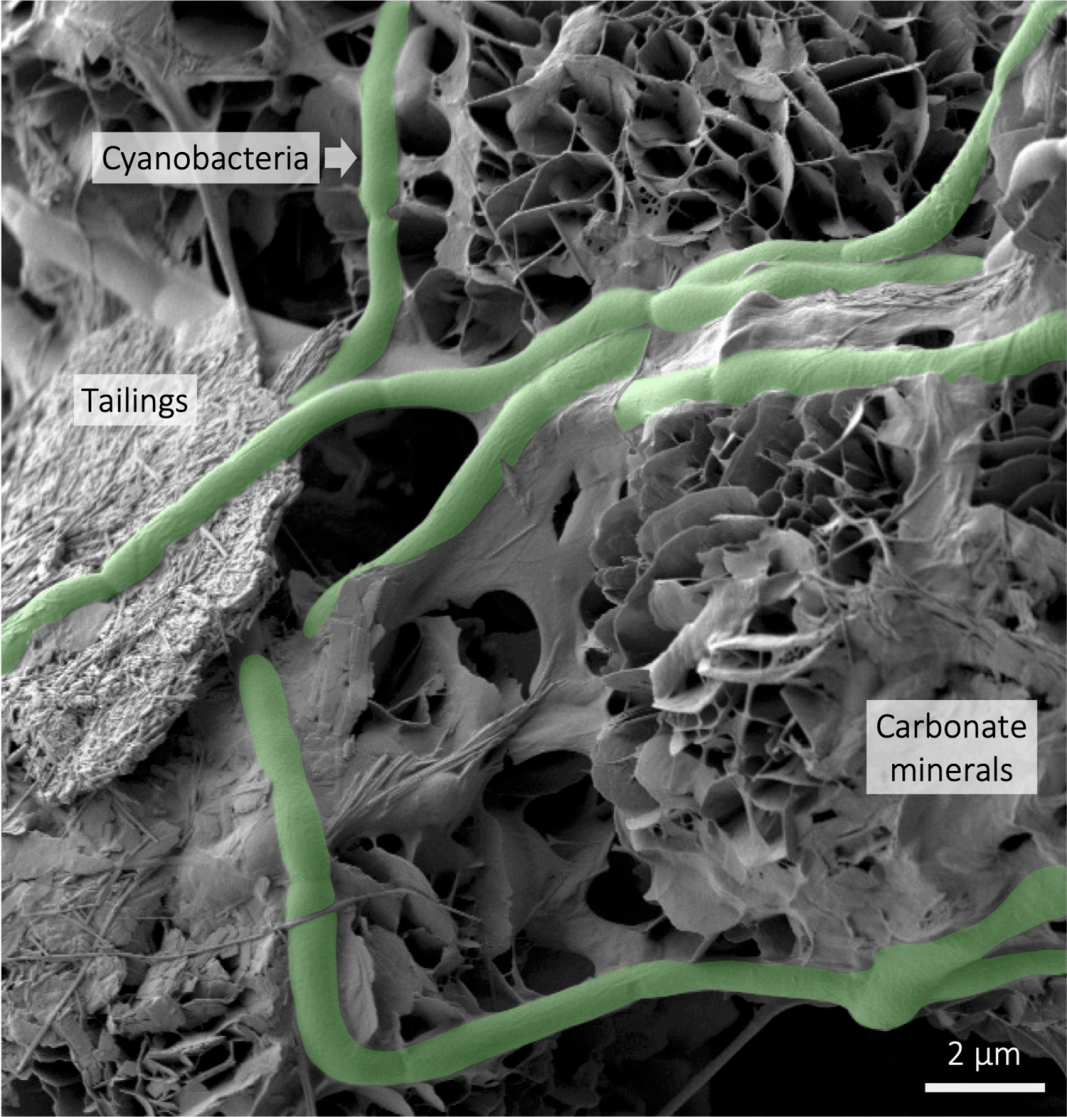
Cyanobacteria (green) precipitating carbonate minerals using tailing mineral weathering by-products [11].

In combination with metal recovery, microbial mineral carbonation can be applied at mine sites, turning them into industrial carbon sinks. Weathering of alkaline mine tailings produces solutions rich in dissolved magnesium and calcium, which react with CO_2_ to form carbonate minerals, thereby providing a pathway for direct air capture of atmospheric CO_2_ [11]. Microbes, including cyanobacteria, ureolytic bacteria, and sulfate-reducing bacteria accelerate carbonate mineral precipitation reactions. Microbes are often found forming carbonate mineral cements in legacy alkaline tailings (Fig 1) [11]. A recent trial of these biogeochemical processes applied to mine wastes from a diamond mine demonstrated that cyanobacteria could contribute to both mineral weathering and subsequent microbial mineral carbonation. Even low rates of microbial mineral carbonation and associated biomass production using cyanobacteria could offset 34% of annual GHG emissions if this process were applied to the entire mine [12]. Emission offsets could be increased through further process optimization to achieve carbon-neutral or potentially carbon-negative mining. These biogeochemical technologies warrant further investigation, given that globally, alkaline and intermediate mine tailings are estimated to have a carbon removal potential of 1 to 5 Gt CO_2_/yr [7,10], a comparable capacity to many of the CDR strategies highlighted by the IPCC. Combining bioleaching, metal recovery, and mineral carbonation of mine tailings gives value to these materials historically deemed industrial “waste.”

Mesoscale experiments have demonstrated microbially mediated CDR, which is ready for testing on a larger scale [12,13]. However, increased funding and industry engagement are needed to advance these technologies and move toward large-scale implementation, from retrofitting legacy sites to designing new mines that pair resource extraction with CDR. This advancement will be achieved through (1) large-scale demonstration projects (e.g., ≥1,000s tonnes) to test microbially mediated CDR on various ore types across a range of climatic conditions using microbial communities, either native or specialized, to each site; (2) optimizing bioleaching and microbial mineral carbonation deployment (e.g., in situ carbonation of tailings versus carbonation in an adjacent wetland) to suit mine design and solve any biogeochemical limitations identified during trials (e.g., nutrient or carbon availability); and (3) having the mining industry engage with governments and stakeholders, including indigenous peoples, environmental consultants, and commercial laboratories, to translate research into standard industry practices. These projects must involve multidisciplinary teams, including geomicrobiologists, geochemists, geologists, metallurgists, and mining engineers, and be jointly funded by the mining industry, private companies interested in acquiring carbon credits, and governments committed to reducing GHG emissions. This green transition will require more rigorous GHG emission policies and more attractive carbon pricing incentives to encourage the implementation of microbially mediated CDR technologies. The mining industry has a unique opportunity to play a significant role in the future of green energy by working toward carbon-neutral mining of critical minerals.
